# Educational Quality and Reliability of YouTube Content Related to Musculoskeletal Ultrasound

**DOI:** 10.5152/ArchRheumatol.2025.25038

**Published:** 2025-09-01

**Authors:** Selcuk Akkaya, Gonca Saglam Akkaya

**Affiliations:** 1 Department of Radiology, Karadeniz Technical University School of Medicine, Trabzon, Türkiye; 2 Department of Physical Medicine and Rehabilitation, Karadeniz Technical University School of Medicine, Trabzon, Türkiye

**Keywords:** Arthritis, inflammatory rheumatic disorders, rheumatology

## Abstract

**Background/Aims:**

YouTube’s growing popularity as an educational resource for musculoskeletal ultrasound (MSKUS) raises questions about its potential to supplement medical education. This study evaluates MSKUS-related YouTube content comprehensively to determine its potential as a supplementary tool in medical education.

**Materials and Methods:**

A cross-sectional analysis was performed on 151 YouTube videos related to MSKUS. Video characteristics and viewer interaction metrics were recorded. Video popularity was quantified using the Video Power Index. The Global Quality Score (GQS), the Quality Criteria for Consumer Health Information (DISCERN), and the Medical Quality Video Evaluation Tool (MQ-VET) were employed to assess the educational value and quality of the videos. Video reliability was evaluated using the Journal of the American Medical Association (JAMA) Benchmark Criteria.

**Results:**

The most frequent MSKUS topic covered was shoulder ultrasound (29.8%), primarily focusing on anatomical landmarks (38.7%). Educational quality assessment indicated that 40.4% of videos were classified as low quality by the GQS. DISCERN rated 43.7% of videos as “very poor” quality, whereas MQ-VET scored 25.8% as average quality. The *JAMA* criteria indicated that 69.5% of the videos provided only partially sufficient information. No videos cited clinical guidelines, 24.5% provided references, and 18.5% included captions. Academic sources demonstrated significantly higher quality (DISCERN: *P* = .018; JAMA: *P* = .015; MQ-VET: *P* = .009). Videos with captions and references/citations demonstrated significantly higher GQS, DISCERN, JAMA, and MQ-VET scores (all *P* < .001). Diagnostic videos had higher GQS (median 3 vs. 2; *P* = .021) and JAMA scores (median 2.5 vs. 2; *P* = .032) compared to injection videos.

**Conclusion:**

This study highlights the inconsistent quality of YouTube-based MSKUS educational content. While academic and well-referenced videos are of high quality, unvetted content often lacks accuracy, making uncurated YouTube videos unreliable for clinical learning. It is recommended that educators guide learners toward content from academic institutions or highly engaged videos with cited guidelines/sources. Standardized guidelines are crucial for integrating trustworthy YouTube MSKUS content into medical curricula.

**Main Points**

- Musculoskeletal ultrasound (MSKUS) videos on YouTube are often of low quality and partially reliable.
- Despite its potential, the inconsistent quality of YouTube’s MSKUS content requires careful evaluation.
- The present study indicates a need for better guidelines and content, especially from academic sources, to improve YouTube’s MSKUS videos.

## Introduction

Ultrasound (US) stands out as a highly efficient and rapid imaging modality for assessing the musculoskeletal system. Its portability and affordability, coupled with dynamic analysis capabilities, make it a valuable tool for clinical practice. US offers several key benefits, including its noninvasive nature and lack of ionizing radiation, leading to its high acceptance among clinicians and patients. Real-time visualization of needles and anatomical structures also suggests that US is an excellent modality for guiding diagnostic and therapeutic interventions.^1^ Musculoskeletal US (MSKUS) has become an increasingly important diagnostic tool in various medical specialties, including rheumatology, physical medicine and rehabilitation, orthopedics, and sports medicine.^2^ As the demand for MSKUS expertise grows, healthcare professionals and students seek accessible and comprehensive educational resources to enhance their knowledge and skills in this field.^3^

The widespread accessibility of the Internet has led to a surge in the number of individuals seeking medical information online, with video-sharing platforms emerging as significant sources of visual health-related content. YouTube has appeared to be a popular platform for medical education, offering a vast array of video content on various healthcare topics.^4^ Although experts contribute a substantial amount of information, the platform’s open nature, which does not verify the credentials of content creators, means that inaccurate or non-expert information can also be readily found.^5,6^

Recognizing YouTube’s growing influence as a medical information resource for the public, there has been a corresponding increase in research focused on evaluating the quality of information available on the platform. However, the quality and reliability of educational content on YouTube can vary significantly, raising concerns regarding the accuracy and completeness of the information presented. Prior studies have generally found that while YouTube offers a vast amount of medical information, the overall quality of its content is often unsatisfactory, with a significant proportion of videos containing biased or poor-quality information.^7,8^

YouTube’s accessibility, user-friendly interface, and diverse content make it an attractive option for learners seeking information about MSKUS techniques, interpretations, and applications. As healthcare professionals increasingly turn to online resources for continuing education and skill development, it is crucial to evaluate the effectiveness and limitations of YouTube as a source of information and education in MSKUS.^9^

The existing literature on MSKUS videos available on YouTube presents conflicting results and focuses on limited aspects, failing to offer a comprehensive evaluation of both their educational quality and reliability for professional learning. One study reported a higher proportion of moderate to high-quality videos,^10^ while another highlighted their poor reliability,^11^ though this was based on a modified and shortened Quality Criteria for Consumer Health Information (DISCERN) scale that included only 5 assessment questions. This inconsistency points to a significant gap in the understanding of YouTube’s true utility and potential drawbacks for professional MSKUS training.

To address inconsistencies in YouTube-based MSKUS content, this study aimed to provide specific recommendations for clinicians and educators. Four validated assessment tools were employed to analyze content quality, incorporating viewer interaction metrics, audio-visual quality, and the presence of captions, references, and guidelines. This comprehensive approach allowed for a nuanced understanding of content reliability and popularity. Furthermore, quality variations were investigated based on video sources and content type (diagnostic vs. injection procedures) to identify more trustworthy sources of information.

## Methods

### Study Design and Data Collection

This cross-sectional study conducted YouTube searches (https://www.youtube.com/) during March2025. Primary search terms, “musculoskeletal ultrasound,” “articular ultrasound,” and “joint ultrasound,” were selected to maximize retrieval breadth using terminology most accessible to diverse audiences (clinicians and trainees). The full term “musculoskeletal ultrasound” was prioritized over abbreviations (e.g., MSKUS or musculoskeletal US), as pilot testing revealed abbreviated forms yielded fewer results and fragmented the dataset due to inconsistent creator usage in titles or descriptions. This approach aligned with clinical terminology in established guidelines such as the European League Against Rheumatism (EULAR) recommendations.

The search results were sorted according to relevance using the default settings of the website. All computer histories and cookies were cleared to avoid restrictions based on user history. The resulting videos were added to a YouTube playlist on a specific date to maintain consistency in ranking.

Videos were excluded if they were irrelevant, did not use US in the procedure, were non-English speaking or lacked English captions, were advertisements, exceeded 1 hour in duration, were duplicated, or were non-speech music videos. Data collection included US and clinical content, video metrics including days on YouTube, video length, count of views, likes, dislikes, and comments. The video sources were categorized as individuals, academic institutions, or other institutions. Caption availability, use of animations/illustrations, and inclusion of references/citations, MSKUS limitations, and clinical guidelines were also recorded.

Two authors, a radiologist and physiatrist, independently screened the first 100 videos for each search term. This cutoff was selected based on established search engine behavior literature demonstrating that >90% of user engagement occurs within the first 20 results,^12^ with a sharp decline thereafter.^13^ Screening 100 videos (equivalent to 5 pages of standard YouTube results) ensured coverage of content with the highest potential visibility to users. This approach aligns with common methodologies in online health content evaluation,^14,15^ which account for the well-documented pattern of diminishing user engagement beyond initial search pages.

### Audio and Visual Quality

Audio quality was evaluated using a 5-point Likert scale according to the Medical Quality Video Evaluation Tool (MQ-VET).^16^ Visual quality was categorized into 2 resolution ranges based on YouTube’s available settings: standard definition (144p-720p) and high definition (1080p-4K).

### Viewer Interaction

The like ratio (likes × 100 / (likes + dislikes)), view ratio (number of views / number of days since upload × 100%), and Interaction Index (likes - dislikes / total number of views × 100) were calculated as measures of viewer interaction.^17^

### Video Popularity

The impact and popularity of the videos were determined using the Video Power Index (VPI) (like ratio × view ratio/100), with higher scores indicating greater popularity.^18^

### Video Quality, Reliability, and Educational Value

The assessment of video quality and educational value was conducted using 3 scoring systems: DISCERN, Global Quality Score (GQS), and the MQ-VET.

DISCERN comprises 15 questions, each worth 5 points, totaling 15-75 points. The assessment comprised 3 sections: the initial 8 items focused on evaluating the reliability of the information, followed by 7 questions detailing the specific treatment characteristics. Higher scores indicated superior information quality. Applying the DISCERN criteria in this study, the analyzed videos were categorized into 5 quality levels: excellent (63-75 points), good (51-62 points), average (39-50 points), poor (27-38 points), and very poor (16-26 points).^19^

The GQS, introduced by Bernard et al,^20^ was employed to evaluate the instructive quality of each video, including its content quality, flow, and ease of use from a patient perspective. This instrument utilizes a 5-point Likert scale, where a score of 1 represents the lowest quality and a score of 5 signifies excellent quality of content. Videos with GQS scores of 4-5 were defined as high quality, a score of 3 as moderate quality, and scores of 1-2 as low quality.^20^

The MQ-VET is a standardized instrument designed to assess the quality and reliability of medical information presented in videos. It achieves this by offering a structured way to evaluate crucial aspects like the accuracy of the information, the expertise of the presenter, and the clarity of the content. The tool has 4 parts, each addressing different aspects with varying numbers of questions: Part 1 has 5 questions, Part 2 has 4, Part 3 has 3, and Part 4 has 3, making a total of 15 questions for the entire tool. All questions are scored on a 5-point Likert scale, ranging from 1 for “Strongly Disagree” to 5 for “Strongly Agree.” The total score, which can be a maximum of 75 points, is calculated by summing the scores from all the questions.^16^ Medical Quality Video Evaluation Tool scores were categorized into 5 quality levels, adopting the methodology of the DISCERN scale described above, owing to the similarity in their scoring systems.

The Journal of the American Medical Association (JAMA) scoring system, a recognized tool for evaluating health-related website information, consists of 4 criteria: “Authorship, Attribution, Disclosure, Currency.” Each criterion was scored as either 0 (not meeting the desired criteria) or 1 (meeting the desired criteria). The scale ranged from 0 to 4, with higher scores indicating better information quality. Following the JAMA methodology, a score of 4 indicated completely sufficient data within the videos, whereas scores of 2 or 3 corresponded to partially sufficient data. Videos that received a score of 0 or 1 were classified as having insufficient data.^21^

To ensure reliability, the audio quality scores and DISCERN, GQS, MQ-VET, and JAMA scores from the 2 physicians’ independent assessments were averaged for subsequent analysis.

This study exclusively utilized publicly accessible YouTube videos and did not involve any human participants or animals. Therefore, in accordance with established practices for similar studies analyzing publicly available online content, formal ethical approval was not deemed necessary. No informed consent was required due to the design of the study which did not include human participants.

#### Statistical Analysis

All statistical analyses were conducted using SPSS version 23.0 (IBM SPSS Corp.; Armonk, NY, USA). The Kolmogorov-Smirnov test was used to check the normality of data distribution. Descriptive analyses were presented using mean ± SD and medians (interquartile range [IQR]) for continuous variables and numbers or percentages for categorical variables. The Mann–Whitney *U* test was employed to compare 2 independent groups. The Kruskal–Wallis test was performed to compare more than 2 independent groups. Pairwise comparisons were performed using the Mann–Whitney *U* test with Bonferroni correction if a significant difference was found in the Kruskal–Wallis test. Correlation analysis was carried out using the Spearman test. Correlation coefficient interpretation followed conventional thresholds: 0.00-0.49 (weak positive), 0.5-0.69 (moderate positive), 0.7-0.89 (strong positive), and 0.9-1 (very strong positive) linear relationships.^22^ The inter-rater agreement was determined with Cohen’s kappa coefficient. The Cohen’s kappa coefficient values ≤ 0, 0.01-0.2, 0.21-0.4, 0.41-0.6, 0.61-0.8, and 0.81-1 indicate no agreement, none to a slight, fair, moderate, substantial, and almost perfect agreement, respectively.^23^ A *P*-value less than .05 was considered statistically significant.

### Results

#### Video Selection

**Figure 1.**
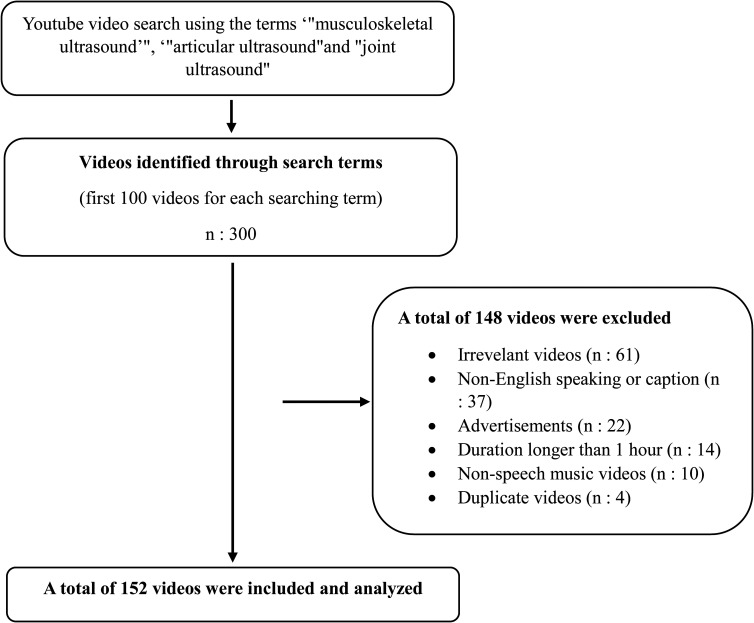
Flowchart of the video search and screening process. figure 1 correction: a total of 149 videos were excluded, irrevelant videos (n:62), a total of 152 videos were included and analyzed.

A total of 300 videos were screened. Of the 149 videos excluded, 62 were irrelevant, 37 had non-English speaking content or captions, 22 were advertisements, 14 had a duration longer than 1 hour, 10 were non-speech music videos, and 4 were duplicates. A final sample of 151 videos were included for analysis (Figure 1).

#### Video Characteristics

The most frequent MSKUS topic was shoulder ultrasound (29.8%), followed by elbow, knee, and ankle/foot ultrasound (14.7% each). Less common topics included temporomandibular joint (0.6%, 1 video) and sacroiliac joint ultrasound (3%, 5 videos). The most common clinical content focused on anatomical landmark assessment (38.7%). Video sources were categorized as individual (44.4%), academic institution (21.9%), and other institution (33.7%). The median number of days on YouTube was 730 (IQR, 365-1460). The median length of the videos was 4.88 (IQR, 1.52-10) minutes. Two videos used animations as a presentation method. Captions were provided in only 18.5% (n = 28) of videos. References/citations were included in 24.5% (n = 37) of the videos, while no videos cited any clinical guidelines.

#### Audio and Visual Quality

Mean audio quality was 4.81 ± 0.50 (median: 5). Assessment of visual quality revealed that 86.8% (n = 131) of the videos were available in high definition, while 13.2% (n = 20) were limited to standard definition.

The main characteristics of the videos are demonstrated in Table 1.

#### Viewer Interactions

The median number of views and likes was 8354 (IQR, 2385-22 758) and 73 (IQR, 27-243), respectively. A single video received 2 dislikes. The median Interaction Index score and view ratio were 1.44 (IQR, 0.5-2) and 1278 (IQR, 233.51-2103), respectively (Table 2).

#### Video Popularity, Quality, and Reliability

The median VPI was 1278 (IQR, 224.34-2103). The median GQS, DISCERN, JAMA, and MQ-VET scores were 3 (IQR, 2-4), 31 (IQR, 22-52), 2 (IQR, 2-3), and 45 (IQR, 36-63), respectively. Cohen’s kappa score representing interobserver agreement was 0.885 (*P* < .001) for the GQS score, 0.829 (*P* < .001) for the DISCERN score, 0.843 (*P* < .001) for the MQ-VET score, and 0.847 (*P* < .001) for the JAMA score (Table 2).

**Figure 2.**
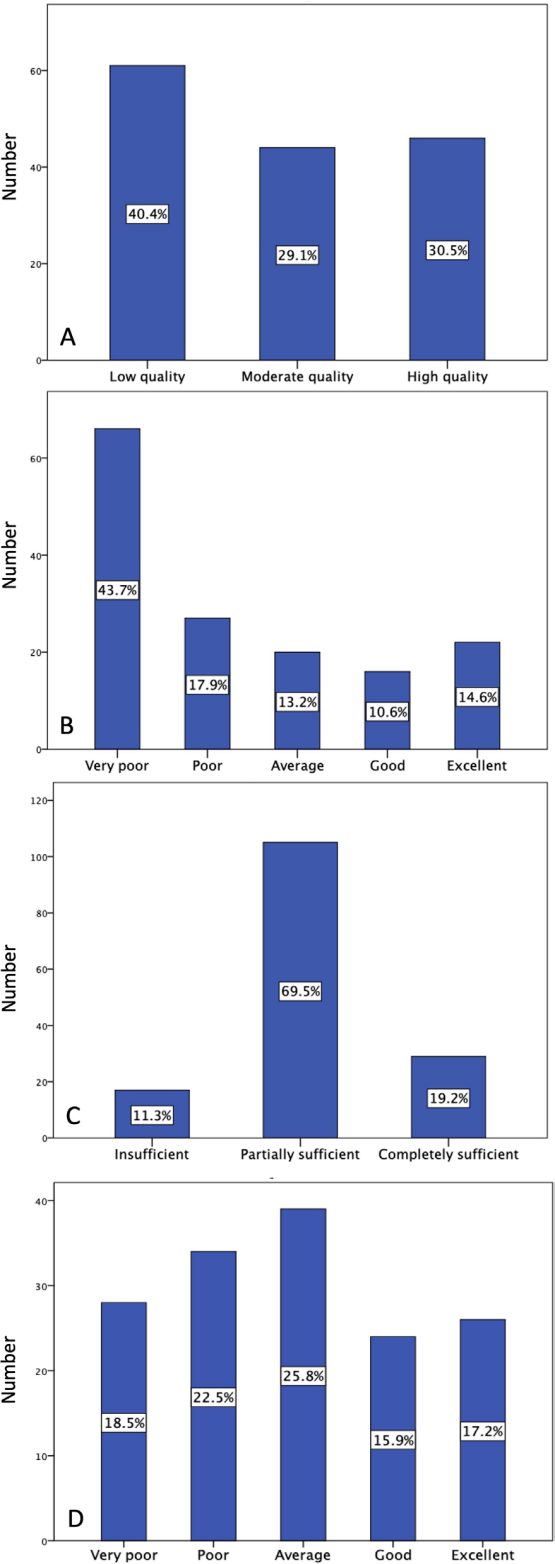
Video distribution according to Global Quality Score (A), DISCERN (B), Journal of the American Medical Association (C), and Medical Quality Video Evaluation Tool (D) scores.

The distribution of videos by GQS, DISCERN, JAMA, and MQ-VET scores is illustrated in Figure 2. Based on GQS, 40.4% of videos were classified as low quality, 29.1% as moderate quality, and 30.5% as high quality. According to DISCERN scores, most videos were rated as very poor quality (43.7%), followed by poor quality (17.9%). Only 14.6% of videos were classified as excellent quality according to DISCERN. The JAMA score classification indicated that most videos (69.5%) provided partially sufficient information. Based on MQ-VET scores, the quality distribution of the videos revealed that 25.8% were categorized as average, while 22.5% were identified as poor quality.

The items receiving the lowest average scores on the DISCERN, JAMA, and MQ-VET scales were, respectively: “description of the risks of each treatment” for DISCERN (1.22 ± 0.74), “references and sources” for JAMA (0.19 ± 0.39), and “concerns about advertising and potential conflicts of interest” for MQ-VET (1.15 ± 0.55). Only 3 videos discussed MSKUS limitations (operator dependency and acoustic shadowing).

DISCERN, JAMA, and MQ-VET scores were higher in academic institution videos than in individual videos (*P* = .018, *P* = .015, and *P* = .009, respectively). No significant differences were observed in the pairwise comparisons between academic institution videos and other institution videos, and between other institution videos and individual videos. There was no statistically significant difference among video groups in terms of video length, number of views, number of likes, number of comments, Interaction Index, like ratio, view ratio, VPI, and GQS (Table 3).

Table 4 presents clinical content analysis. Videos focusing on diagnostic applications of MSKUS were significantly longer than those covering ultrasound-guided injections (*P* < .001). Injection videos garnered higher median views (*P* = .023) with fewer comments (*P* = .027) and lower interaction indexes (*P* < .001). Diagnostic videos demonstrated higher GQS (*P* = .021) and JAMA scores (*P* = .032). No significant differences emerged in DISCERN or MQ-VET scores between these categories.

Table 5 shows video properties according to caption availability, visual quality, and inclusion of references/citations. Videos with captions exhibited significantly higher educational quality across all assessment tools: GQS, DISCERN, JAMA, and MQ-VET (all *P* < .001). Similarly, videos including references/citations scored higher on GQS, DISCERN, JAMA, and MQ-VET scales (all *P* < .001). Referenced videos also attracted more views (*P* = .029), likes (*P* = .011), and comments (*P* = .009) with higher VPI scores (*P* = .009). Visual quality showed no significant association with any metric.

The results of Spearman correlation analyses revealed significant associations among several video characteristics, popularity, quality, and reliability assessment scales. Video length correlated weakly with higher GQS (rho = 0.249, *P* = .002), JAMA (rho = 0.254, *P* = .002), and MQ-VET scores (rho = 0.171, *P* = .036). Likes, comments, and VPI demonstrated weak-to-moderate positive correlations with all quality and reliability scales (GQS, DISCERN, JAMA, MQ-VET). Strong intercorrelations existed among assessment tools, particularly between DISCERN and MQ-VET (rho = 0.872, *P* < .001) and JAMA and GQS (rho = 0.828, *P* < .001). Audio quality and days on YouTube showed no significant correlations with any scale (Table 6).

## Discussion

The increasing demand for MSKUS training opportunities has led healthcare professionals to explore various educational resources, including online platforms such as YouTube. The present study revealed a generally low level of educational quality and partially sufficient videos that could potentially lead to misinformation. The results are consistent with previous research examining YouTube content across various medical specialties.^24-27^ Videos produced by academic institutions exhibited superior quality and reliability compared to those from individual sources. Furthermore, content focusing on diagnostic procedures was notably longer and demonstrated higher educational quality, as assessed by the GQS and JAMA criteria, although videos related to injection procedures garnered more views. Critically, videos featuring captions or references/citations exhibited superior quality across all assessment tools (GQS, DISCERN, JAMA, MQ-VET) and attracted greater viewer engagement (views, likes, comments, VPI).

Several studies have proposed methods for evaluating the quality of online health information, including the use of tools such as the DISCERN and JAMA benchmark criteria, with contradictory results. Additionally, VPI is recommended for a more comprehensive assessment of video popularity.^28^ Some previous studies observed that VPI decreased as video quality improved.^6,29^ A study by Staunton et al^31^ on scoliosis revealed an inverse relationship between information quality and view count.^30^ Other studies on influenza pandemics, spondyloarthritis, and rheumatoid arthritis found no statistically significant differences in audience interaction metrics between useful and misleading videos.^31,32^

In contrast to prior investigations, the current findings indicated a positive correlation between video quality and popularity. It is suggested that this divergence may be due to the distinct composition of the inferred YouTube audience, which likely comprised a higher proportion of healthcare professionals compared to previous studies. Their enhanced prior knowledge likely led to a preference for and greater interaction with higher-quality content, resulting in increased online interactions and popularity metrics for these videos.

The implications of low-quality or unreliable US information on YouTube are significant. For patients seeking to comprehend diagnostic procedures or potential treatments involving US, exposure to inaccurate or incomplete information can lead to unrealistic expectations, anxiety, and suboptimal decision-making. For healthcare professionals, particularly those training or new to US techniques, reliance on unverified online resources could result in the adoption of suboptimal or even harmful practices.^33,34^

A few studies have investigated YouTube videos, including those related to US practices, using both diagnostic and injection techniques. Cüzdan et al^10^ assessed the educational quality and reliability of 58 MSKUS-related YouTube videos similar to the present study. Consistent with the current findings, the modified DISCERN tool further underscored the poor reliability of the content, with a total median value of 2.^11^ Another study found that 60% of MSKUS videos were rated as high and moderate quality according to the modified DISCERN scores, whereas excellent, good, and average videos were identified at a total rate of 38.4%, as evaluated by DISCERN scores. Using different criteria, rater variability, and differences in the video samples may result in this discrepancy.^12^ Additionally, it is important to note that the source of a video may influence its content, perspective, and potential biases. It was observed that DISCERN, JAMA, and MQ-VET scores were higher for videos originating from academic institutions.

An investigation was conducted to ascertain the utility and quality of video content on YouTube for US-guided breast biopsy. The findings indicated that a minority (13.7%) of the analyzed videos were very useful, while a larger proportion (41.2%) was classified as useful. Notably, a substantial majority (85.7%) of the highly beneficial videos were produced by physicians or hospital entities, and the DISCERN scores were significantly elevated in the very useful video cohort. However, videos uploaded by non-medical individuals received more likes and comments.^35^ Cho et al^37^ evaluated the usefulness and quality of YouTube in performing ultrasound-guided brachial plexus block. The findings revealed that academic, manufacturing, and educational videos demonstrated superior accuracy and reliability compared with individual videos.^36^ Another cross-sectional study assessed the educational quality of ultrasound-guided dry needling videos, and the mean DISCERN and JAMA scores indicated low quality.^38^

The findings of this study align with existing research on the use of US, highlighting the inconsistent quality of online resources for healthcare procedures. These results suggest a risk of misinformation being spread through freely accessible video platforms. The inherent accessibility of platforms like YouTube, combined with the lack of a formal peer-review process, likely contributes to the scarcity of high-quality educational videos. The variability in the sources of these videos appears to significantly influence their content quality.

The investigation of key quality characteristics in MSKUS videos revealed significant deficiencies, exceeding those reported in similar studies. Notably, none of the analyzed videos cited guidelines such as the EULAR recommendations, and fewer than one-quarter provided references. The overwhelming majority consequently appear to rely primarily on presenters’ personal expertise without explicit linkage to established standards or supporting literature. This gap may originate from platform limitations (e.g., technical challenges in displaying citations during videos) or heterogeneous creator motivations. Captions were provided in only 28 videos (18.5%), indicating limited accessibility support for viewers and excluding hearing-impaired learners. Only 3 videos acknowledged fundamental MSKUS limitations. Furthermore, the lowest-scoring items were ‘description of treatment risks’ on the DISCERN and ‘advertising/conflict of interest disclosure’ on the MQ-VET scale. In contrast, the technical quality of the videos, including both audio and visual aspects, was generally high.

The methodology of this study, which includes the use of multiple assessment tools and independent evaluations by experts, enhances the reliability of the findings. The video content was categorized into various topics, such as video sources and clinical procedures. This categorization provides valuable context for understanding the nature of MSKUS content on YouTube. The inter-rater agreement further adds credibility to the assessments.

This study has several limitations. Firstly, the cross-sectional design provides only a snapshot of YouTube content at a specific time, and the dynamic nature of online data means longitudinal studies are needed to track changes in content quality and trends. Secondly, the study focused solely on English-language videos, which may not represent the full global landscape of MSKUS education on YouTube. The search strategy prioritized full terminology over abbreviations (e.g., MSKUS). While this approach aligned with the EULAR clinical lexicon, relevant content may have been missed. Finally, focusing solely on YouTube neglects content on other online platforms.

As a result, the overall quality of YouTube videos designed to enhance healthcare professionals’ practical skills in this area was found to be largely low quality. These findings have important implications for both content creators and consumers of MSKUS educational videos on YouTube. For content creators, particularly those affiliated with academic institutions, there is an opportunity to improve the quality of MSKUS videos by adhering to established guidelines for medical education and information dissemination. For viewers, the study underscores the importance of critically evaluating video content and cross-referencing information with peer-reviewed sources. Viewers should prioritize content from accredited institutions/professional societies, actively seek cited sources in descriptions, and cross-verify information against peer-reviewed literature and official guidelines before applying it clinically.

The divergence between diagnostic and injection MSKUS videos underscores YouTube’s dual role as a quick-reference tool and a potential educational supplement. While injection videos dominate viewership, their educational limitations necessitate cautious use. Future content should bridge this gap by embedding diagnostic rigor into procedural guidance, ensuring both efficiency and evidence-based reliability. In addition, future studies could explore content in multiple languages and include additional video sources, such as Vimeo or MedTube, to provide a more comprehensive understanding of the educational potential of similar platforms worldwide. Structured, affordable online programs that follow validated guidelines are needed to ensure consistency and accuracy.

While YouTube offers a vast, accessible repository of MSKUS educational content, its variable quality necessitates careful evaluation and precludes its use as a primary substitute for formal training or clinical experience. To leverage its potential as a supplementary resource, educators and learners should prioritize content from established academic institutions and favor videos featuring captions, references/citations, and higher viewer engagement, as these characteristics correlate with improved quality and reliability. Academic institutions are crucial in enhancing the quality of YouTube-based MSKUS content by leveraging their expertise to produce accurate, comprehensive, and well-referenced videos. Crucially, developing standardized guidelines for curating and integrating trustworthy YouTube MSKUS content into curricula is essential. Future research must focus on enhancing content quality and establishing effective, validated strategies for incorporating online video resources into formal medical education.

## Figures and Tables

**Table 1.** Content Evaluation and Characteristics of the Videos

		**n**	**%**
Ultrasound content*	Shoulder	49	29.8
	Elbow	24	14.7
	Wrist/hand	17	10.3
	Hip	20	12.2
	Knee	24	14.7
	Ankle/foot	24	14.7
	Sacroliac joint	5	3
	Temporomandibular	1	0.6
Clinical content*	Probe holding and scanning	113	33.6
	Anatomical landmarks	130	38.7
	US diagnosis	60	17.9
	US-guided injections	33	9.8
Video Source	Individual	67	44.4
	Academic Institution	33	21.9
	Other Institution	51	33.7
Visual quality	Standard definition:144p-720p	20	13.2
	High definition: 1080p-4K	131	86.8
Caption availibility		28	18.5
Inclusion of references/citations		37	24.5
		**Mean ± SD**	**Median (IQR)**
Video metrics	Days on YouTube	1262.72 ± 1430.84	730 (365-1460)
	Length (minutes)	7.92 ± 8.76	4.88 (1.52-10.00)
**Audio quality**		4.81 ± 0.50	5 (5-5)

IQR, interquartile range; n, number; US, ultrasound.

*More than 1 option may apply.

**Table 2.** Interaction Parameters, Video Power Index, Global Quality Scale, DISCERN, and Journal of the American Medical Association Scores of the Videos

	Mean ± SD	Median (IQR)
Viewer interactions		
	View	18 477.08 ± 27 354.32	8354 (2385-22 758)
	Likes	241.13 ± 428.07	73 (27-243)
	Dislikes	0.01 ± 0.16	0 (0-0)
Comments	6.91 ± 12.54	2 (0-7)
Interaction Index	1.72 ± 2.09	1.44 (0.5-2)
Like Ratio	97.97 ± 14	100 (100-100)
View Ratio	1671.02 ± 1712.82	1278 (233.51-2103)
Video Power Index	1670.92 ± 1712.89	1278 (224.34-2103)
Global Quality Scale	2.92 ± 1.10	3 (2-4)
DISCERN	36.34 ± 18.93	31 (22-52)
JAMA	2.53 ± 0.94	2 (2-3)
MQ-VET	47.79 ± 16.08	45 (36-63)

IQR, interquartile range; JAMA, Journal of the American Medical Association; MQ-VET, Medical Quality Video Evaluation Tool.

**Table 3.** Video Properties According to Video Sources

	Individual Median (IQR)	Academic Institution Median (IQR)	Other Institution Median (IQR)	P*
Length (minutes)	4.27 (1.25-9.78)	4.85 (1.90-8.15)	6.42 (2.08-10)	.445
Views	5813 (3948-14 112)	12 498 (962-21 824)	10 376 (1179-52 643)	.630
Likes	71 (29-204)	156 (28.50-199)	73 (6-463)	.974
Comments	1 (0-8)	4 (1-6)	1 (0-8)	.736
Interaction Index	1.61 (0.50-2.27)	1.39 (0.60-1.73)	1.27 (0.39-1.89)	.682
View ratio	990.51 (397-2328)	1917 (150.68-1917)	1388 (91.32-3561)	.991
Video Power Index	990.51 (397-2328)	1917 (150.68-1917)	1388 (91.32-3561)	.991
Global Quality Scale	3 (2-3)	3 (2.50-4)	3 (2-4)	.126
DISCERN	**30 (22-41)**	**44 (25-60.50)**	**29 (19-61)**	**.018**
JAMA	**2 (2-3)**	**3 (2-4)**	**2 (2-3)**	**.015**
MQ-VET	**44 (35-55)**	**55 (45-67)**	**50 (36-68)**	**.009**

IQR, interquartile range; JAMA, Journal of the American Medical Association; MQ-VET, Medical Quality Video Evaluation Tool.

*Kruskal–Wallis test.

**Table 4.** Video Properties According to Clinical Content

	Diagnostic Procedures Median (IQR)	US-Guided Injections Median (IQR)	P*
Length (minutes)	**6.15 (2.06-14.13)**	**1.77 (1.49-4.3)**	**<.001**
Views	**5800 (2100-21 000)**	**9500 (5000-48 000)**	**.023**
Likes	96 (27-308.75)	67 (26-199)	.268
Comments	**3 (0-9.5)**	**1 (0-3.5)**	**.027**
Interaction Index	**1.63 (0.94-2.19)**	**0.40 (0.29-0.7)**	**<.001**
View Ratio	1433.50 (210.74-3287)	730.59 (428.6-1572.5)	.252
Video Power Index	1433.50 (210.74-3287)	730.59 (428.6-1572.5)	.252
Global Quality Scale	**3 (2-4)**	**2 (2-4)**	**.021**
DISCERN	32 (24.5-54)	25 (19.5-46)	.092
JAMA	**2.5 (2-3)**	**2 (1.5-3)**	**.032**
MQ-VET	47 (36-64)	45 (32-62.5)	.486

IQR, interquartile range; JAMA, Journal of the American Medical Association; MQ-VET, Medical Quality Video Evaluation Tool; US, ultrasound;.

*Mann–Whitney *U* test.

**Table 5.** Video Properties According to Caption Availability, Visual Quality, and Inclusion of References/Citations

	Caption Availability Median (IQR)			Visual Quality Median (IQR)			Inclusion of References/Citations Median (IQR)		
	None	Included	P*	144p-720p	1080p-4K	P*	None	Included	P*
Length (minutes)	4.86 (1.5-10)	6.2 (2.13-9.98)	.226	16.21 (1.82-28.93)	4.88 (1.52-9.38)	.054	4.85 (1.52-9.78)	6.28 (2.55-18.62)	.058
Views	8700 (3175-22 500)	5300 (637-22 000)	.357	5207 (1125-51 250)	8500 (2500-22 000)	.932	**5800 (1500-22 000)**	**18500 (4800-47 750)**	**.029**
Likes	79 (26.75-233-25)	71 (26-374)	.971	63.5 (16.5-772.5)	73 (27-243)	.932	**71 (17-226)**	**199 (67.25-487.25)**	**.011**
Comments	1 (0-7)	5 (0-10)	.052	1 (0-3.75)	3 (0-7)	.188	**1 (0-6)**	**5.5 (1-12)**	**.009**
Interaction Index	1.27 (0.5-2)	1.66 (0.89-2.25)	.059	1.11 (0.66-2.24)	1.44 (0.5-2)	.926	1.39 (0.5-1.89)	1.71 (0.83-2.24)	.107
View ratio	1333 (237-2103)	1236 (150.68-3371.5)	.991	1249.5 (106.16-2056.5)	1278 (256.16-2438)	.705	**990.5 (210-1917) **	**1917 (819-3287)**	**.009**
Video Power Index	1333 (237-2103)	1236 (150.68-3371.5)	.991	1249.5 (106.16-2056.5)	1278 (256.16-2438)	.705	**990.5 (210-1917)**	**1917 (819-3287)**	**.009**
GQS	**3 (2-3)**	**4 (3-4)**	**<.001**	3 (1.25-3)	3 (2-4)	.099	**3 (2-3)**	**4 (4-5)**	**<.001**
DISCERN	**30 (20-45.75)**	**42 (28.5-64)**	**<.001**	28 (11.25-48)	32 (23-54)	.129	**29 (20-41)**	**64 (54.5-70)**	**<.001**
JAMA	**2 (2-3)**	**3 (2-4)**	**<.001**	2 (1.25-3)	2 (2-3)	.208	**2 (2-3)**	**4 (3-4)**	**<.001**
MQ-VET	**44 (32-60)**	**60 (45-70)**	**<.001**	44 (20.5-58.75)	47 (37-64)	.102	**44 (32-55)**	**69 (60.5-70)**	**<.001**

GQS, Global Quality Score; IQR, interquartile range; JAMA, Journal of the American Medical Association; MQ-VET, Medical Quality Video Evaluation Tool.

*Mann–Whitney *U* test.

**Table 6.** Correlations Between Video Characteristics and Interaction parameters, Video Power Index, Global Quality Score, DISCERN, and Journal of the American Medical Association Scores of the Videos

	GQS		DISCERN		JAMA		MQ-VET	
	rho*	*P*	rho*	*P*	rho*	*P*	rho*	*P*
Days on Youtube	−0.117	.154	0.074	.366	−0.086	.293	0.003	.966
Length (minutes)	**0.249**	**.002**	0.114	.164	**0.254**	**.002**	**0.171**	**.036**
Views	**0.234**	**.004**	**0.243**	**.003**	0.123	.133	**0.263**	**.001**
Likes	**0.387**	**<.001**	**0.294**	**<.001**	**0.263**	**.001**	**0.341**	**<.001**
Comments	**0.401**	**<.001**	**0.305**	**<.001**	**0.253**	**.002**	**0.292**	**<.001**
Interaction Index	**0.257**	**.001**	0.130	.111	**0.249**	**.002**	**0.166**	**.041**
Like ratio	−0.065	.425	−0.044	.592	0.000	.997	−0.022	.793
View ratio	**0.423**	**<.001**	**0.317**	**<.001**	**0.246**	**.002**	**0.334**	**<.001**
Video Power Index	**0.423**	**<.001**	**0.317**	**<.001**	**0.246**	**.002**	**0.334**	**<.001**
Audio quality	0.029	.723	0.054	.510	−0.045	.584	0.027	.739
GQS	–	–	**0.734**	**<.001**	**0.828**	**<.001**	**0.704**	**<.001**
DISCERN	**0.734**	**<.001**	–	–	**0.678**	**<.001**	**0.872**	**<.001**
JAMA	**0.828**	**<.001**	**0.678**	**<.001**	–	–	**0.631**	**<.001**
MQ-VET	**0.704**	**<.001**	**0.872**	**<.001**	**0.631**	**<.001**	–	–

GQS, Global Quality Score; JAMA, Journal of the American Medical Association; MQ-VET, Medical Quality Video Evaluation Tool.

*Spearman correlation coefficients.

## Data Availability

The datasets generated or analyzed during the study are available from the corresponding author upon reasonable request.
